# Associations of Plant-Based Foods, Animal Products, and Selected Sociodemographic Factors with Gastroesophageal Reflux Disease Risk

**DOI:** 10.3390/ijerph21121696

**Published:** 2024-12-19

**Authors:** Ahlam El Shikieri, Zakaria Eltahir, Abdulmannan Aman, Mohamad Alhadramy

**Affiliations:** 1 Department of Clinical Nutrition, College of Applied Medical Sciences, Taibah University, Al Madinah Al Munawarah 42313, Saudi Arabia; 2 Department of Clinical Laboratories, College of Applied Medical Sciences, Taibah University, Al Madinah Al Munawarah 42313, Saudi Arabia; zidrees@taibahu.edu.sa; 3 University Medical Center, Faculty of Medicine, Taibah University, Al Madinah Al Munawarah 42313, Saudi Arabia; amamohamed@taibahu.edu.sa; 4 Charitable Medical Care Society, Al Madinah Al Munawarah 42313, Saudi Arabia; alhadraamy@hotmail.com

**Keywords:** GERD, dietary quality, demographic characteristics, animal products, plant-based foods, Saudi Arabia, GERMS

## Abstract

Background: Diet influences the symptoms of gastroesophageal reflux disease (GERD). Plant-based diets rich in vegetables, fruits, legumes, seeds, and nuts may reduce inflammation and improve gut health, while high-fat foods may worsen symptoms. Objective: We examined the association between plant-based and animal-based foods, selected demographic characteristics, and the likelihood of GERD in Al Madinah Al Munawarah, Saudi Arabia. Method: A cross-sectional study using the GerdQ tool assessed the GERD likelihood among 303 adults. Dietary diversity scores were used to assess the quality of their diet. quality. Results: The participants were predominantly women (68.6%) and had low education levels (88.4%). Cereals were the most consumed plant-based foods, while vitamin A-rich fruits and vegetables were the least consumed. There was significant variation in the consumption of legumes, nuts, seeds, and milk and milk products among the GERD groups. The participants with a 50% GERD likelihood had the highest consumption (34.5%), followed by the 89% likelihood group (21.4%) and the 79% likelihood group (14.5%). The lowest consumption of milk and milk products was among those with an 89% GERD likelihood who also consumed more organ meat. In addition, GERD likelihood was inversely associated with age (r = −0.153; *p* = 0.008). The likelihood of GERD was negatively correlated with the intake of legumes, nuts, and seeds (r = −0.163; *p* = 0.005). Furthermore, the intake of cereals and tubers (r = 0.114; *p* = 0.047) and legumes, nuts, and seeds (r = 0.231; *p* = 0.0001) increased significantly with education. Conclusion: GERD prevention programs should target women, those with a low education level, and individuals consuming fewer plant-based foods and more organ meats.

## 1. Introduction

Gastroesophageal reflux disease (GERD) is a prevalent and chronic gastrointestinal disorder that affects many people globally, with an estimated prevalence of 18–28% in North America and 8.7–33.1% in the Middle East [1]. The disease is defined as “A condition which develops when the reflux of stomach contents causes troublesome symptoms (i.e., at least two heartburn episodes per week) and/or complications” [2]. In addition to heartburn, patients might suffer from regurgitation, chest pain, and other potential complications like esophagitis and Barrett’s esophagus [2,3]. The consequences of GERD could be detrimental, leading to a decreased quality of life [4], an increased risk of cardiovascular diseases [5], and mental health conditions such as anxiety [6]. Thus, GERD threatens human health and can worsen the burden on patients and the community [7] by increasing the risk of esophagitis, a hemorrhage, and adenocarcinoma [8].

Recent research has shed light on the potential impact of dietary choices on developing and managing GERD. Evidence suggests a direct relationship between reflux symptoms and fat-rich and fried foods (including organ meat); animal-based products (e.g., meat, dairy, eggs); citric foods (such as low pH foods, acid-forming foods, and sour foods); chocolate; and beverages like tea and coffee [3]. Plant-based food (PBF) dietary patterns have garnered significant research interest among emerging nutritional interventions. A well-structured PBF diet emphasizes the consumption of nutrient-rich plant-derived foods, including raw and cooked vegetables, fruits, legumes (including beans, peas, lentils, and soybeans), and moderate portions of seeds and nuts. The beneficial effects of plant-based diets on GERD symptoms may be attributed to their lower content of reflux-triggering components such as processed foods, oils, and animal products (including eggs) [9], which worsens esophageal reflux [10]. This dietary pattern aligns with a Mediterranean diet, which includes fish and dairy products [3]. This dietary strategy is rooted in its potential to positively influence various aspects of health, including reducing the risk of GERD [3], heart disease, diabetes [9], and obesity [11].

The potential role of PBF diets in GERD symptom management has emerged as a significant area of investigation. Current literature indicates that PBF diets—characterized by abundant intake of fruits, vegetables, whole grains, legumes, and nuts—optimize consumption of nutrient-dense plant materials [9,12]. These dietary patterns may confer multiple therapeutic benefits, including attenuation of inflammatory processes, enhanced antioxidant defense mechanisms, and favorable modulation of gut microbiota composition [11,12,13,14]. As such, a PBF diet can reduce oxidative stress on the esophageal mucosa, which plays a vital role in the pathogenesis of GERD [15]. Moreover, previous studies have shown that people on a plant-only diet, i.e., those who did not consume meat, fish, poultry, dairy products, or eggs, were at a lesser risk for GERD [16,17]. On the other hand, the consumption of red meat and a high-fat diet correlate with increased acid exposure time when compared to a low-fat meal [3].

Further studies have shown that there are sociodemographic differences among GERD patients. For instance, the disease is more prevalent in females than males [7,17,18]. However, other studies have revealed that men were at a higher risk of GERD than women [19]. In addition, younger people are at a lesser risk for GERD than their older counterparts. Thus, advanced age was associated with a higher GERD risk [18]. However, some studies have reported that age did not influence the prevalence of GERD [20]. In contrast, previous studies in Saudi Arabia showed that the individuals who were unmarried and had higher levels of education were more likely to develop GERD compared to those who were married and had lower education levels [21]. There is disagreement concerning the sociodemographic factors and the risk of GERD. Therefore, the current study will further address the association between age, sex, and education and GERD risk.

The current study explored the relationship between plant- and animal-based foods and GERD likelihood as part of the Gastroesophageal Reflux Madinah Study (GERMS) project. The GERMS is a cross-sectional research initiative examining GERD in Al Madinah Al Munawarah, Saudi Arabia. This two-year project started in 2022 and is funded by the Deputyship for Research and Innovation of the Ministry of Education in Saudi Arabia (Grant No.: 442/126). The project aimed to identify the region-specific risk factors and treatment patterns among adults with GERD symptoms. The investigation encompasses a histopathological analysis of gastric biopsies, an evaluation of prescribed therapeutic regimens, and an assessment of mental health, dietary patterns, and nutritional status through validated questionnaires. The findings are intended to establish evidence-based clinical practice guidelines tailored to the local population and potentially inform GERD management strategies in similar demographic contexts. The project is currently in its final data analysis and manuscript preparation phase.

Furthermore, we examined the relationship between the consumption of plant- and animal-based foods and the selected demographic characteristics and the likelihood of GERD. By doing so, we hope to provide valuable insights into the dietary management of GERD and guide advanced research in this area to reduce future GERD complications, such as Barett’s esophagus [2]. It is hypothesized that the likelihood of GERD is increased with lower adherence to plant-based foods, higher consumption of animal-based foods, and selected demographic characteristics. The novelty of our study lies in the fact that it followed a diverse demographic sampling that included men and women from different educational backgrounds and age groups. In addition, this study is culturally relevant; it includes participants from various backgrounds while addressing local dietary habits. This could potentially lead to more effective dietary recommendations and future interventions.

## 2. Materials and Methods

A public announcement targeted adult men and women at the outpatient clinics of the Charitable Medical Care Society and Taibah University Medical Centre in Al Madinah Al Munawarah, Saudi Arabia. The inclusion criteria included adults aged ≥18 years old who had no history of complicated disease conditions such as cancer, liver cirrhosis, or renal failure. Pregnant and lactating women were excluded.

The study’s sample size was calculated based on a previous study conducted in Riyadh, Saudi Arabia. In that study, 1265 participants filled out a questionnaire, and the prevalence of GERD was estimated to be 45.4% [22]. The following equation was used to calculate the minimum sample size:

n = (Z_1−α/2_)^2^ × P (1 − P) ÷ d^2^

where

n = the sample size;
Z_1−α/2_ = 1.64 for a 90% confidence level;
d = the desired margin of error, expressed as a decimal (0.05);
P = the prevalence of the disease is 45.4% (0.454).

Based on this calculation, the minimum required sample size in the current study was 270 participants. To account for the unexpected drop out of participants, 10% was added to the minimum sample size.

Several tools were used to obtain information about demographic characteristics, the likelihood of GERD, and dietary quality and diversity. The details of each tool are listed below. The demographic characteristics included information about age in years (18–28, 29–39, 40–49, 50–59, 60–69, and ≥70), sex, and education level (a low education level included no formal education, as well as primary and secondary schooling; a high education level included university and postgraduate studies). These characteristics were the independent or the predictor variables. The GERMS has several objectives, one of which will be investigated in this paper.

In addition, GERD likelihood was assessed using a previously validated Arabic version of the GerdQ questionnaire [23]. The GerdQ questionnaire (Table S1) demonstrates significant utility as a diagnostic instrument for GERD in primary care settings [24], enabling both initial diagnosis and management protocols without necessitating immediate specialist consultation or endoscopic evaluation [25]. This questionnaire asked six questions about the following categories: the presence of heartburn (how often do you have a burning feeling behind your breastbone), regurgitation (how often do you have stomach contents (liquid or food) moving upwards to your throat or mouth), pain in the center of the upper stomach (how often do you have a pain in the center of the upper stomach), nausea (how often do you have nausea), sleep disturbances (how often do you have a good night sleep because of your heartburn and/or regurgitation), and the intake of over-the-counter medications (how often do you take additional medication for your heartburn and/or regurgitation, other than what the physician told you to take, such as Tums, Rolaids, or Maalox). Symptom frequency was documented by participants on a scale ranging from zero to seven days during the preceding week. The patients who scored 0–2 on the GerdQ have a zero percent likelihood of GERD; those with scores of 3–7 have a 50% likelihood; those with scores of 8–10 have a 79% likelihood; and those with scores of 11–18 have an 89% likelihood of GERD. The latter was considered the study’s outcome variable (dependent variable).

The Food and Agriculture Organization’s Individual Dietary Diversity Score Questionnaire (IDDS) assessed the dietary quality, diversity, and number of food groups consumed (Table S2). A hypothesis-driven method was used to identify the participants’ dietary patterns. The participants were asked about their previous 24 h dietary intake without quantifying the amounts and portion sizes. The participants were requested to systematically list the foods and drinks from the morning to the evening without interruption. Then, they were asked if they consumed any foods and/or drinks between meals and were given hints to help them remember the foods that could be forgotten, such as juices, tea with sugar, coffee, sandwiches, and soft drinks. After they finished, the list was read to them to ensure no information was missing. These methodological procedures were implemented to minimize collection bias, enhance recall accuracy, and validate our hypothesis regarding the differential impacts of plant-based versus animal-based dietary patterns on GERD risk. FAO chose the recall period of 24 h as it is less subject to recall error, less cumbersome for the respondent, and conforms to the recall period used in many dietary diversity studies [26].

The foods were then grouped based on the IDDS into nine plant- and animal-based food groups. The plant-based food groups included cereal-based foods and tubers, dark-green leafy vegetables, vitamin A-rich fruits and vegetables, other fruits and vegetables, and legumes, nuts, and seeds. The groups that belonged to the animal-based groups included organ meat (e.g., liver, kidney, heart), fleshy meat (e.g., beef, chicken, and lamb), fish (fresh, dried, or shellfish), eggs, and milk and dairy products [26]. The current study combined flesh meat and fish as one group for an easy analysis. The score was calculated based on which food groups were consumed throughout the day. A score of (1) was given if the participants answered “Yes” and (0) if they answered “No”; the total number of food groups consumed was then scored. The quality was categorized as ≤3—low, 4–5—medium, and ≥6—high [27]. The dietary diversity score was calculated for each person by the summation of the number of times different food items under each food group were eaten in a day. The plant- and animal-based foods were the predictors or independent variables. Trained nurses assisted in data collection.

### 2.1. Ethical Considerations

This study was approved by the IRB committee at the College of Applied Medical Sciences (certificate number SREC/AMS 2021/100/120/MLT) and the IRB at the General Directorate of Health Affairs in Al Madinah Al Munawarah (number 583). Informed consent was obtained from all the participants involved in this study. Participants were assured of strict data confidentiality and privacy protocols, and were informed of their autonomy to terminate study participation at any point without adverse consequences.

### 2.2. Statistical Analysis

In the current study, the independent variables were diet quality and the intake of a plant-based diet, namely the nine food groups stated by the DDS. GERD probability was stratified into four distinct risk categories: 0%, 50%, 79%, and 89%, serving as the primary outcome measure. The data were analyzed using the Statistical Package for Social Sciences (IBM SPSS Statistics for Windows, Version 26.0. IBM Corp., Armonk, NY, USA). Descriptive statistics were used, including mean, standard deviation, and percentages. A Chi-square test was used to compare the categorical variables. Tukey’s HSD post hoc one-way analysis of variance (ANOVA) was performed to determine the differences among the various GERD likelihoods regarding dietary diversity and demographic characteristics. The Mann–Whitney U test was used for non-parametric tests (e.g., total GerdQ scores). The comparative outcomes of the analysis using the Chi-square test were used for categorizing the continuous data. Pearson’s correlation was used to determine the association between variables. A multiple regression analysis was used to assess the factors associated with the likelihood of GERD. The 95% confidence intervals (CIs) are shown where applicable. The significance level was set to 5%.

## 3. Results

**Figure 1:**
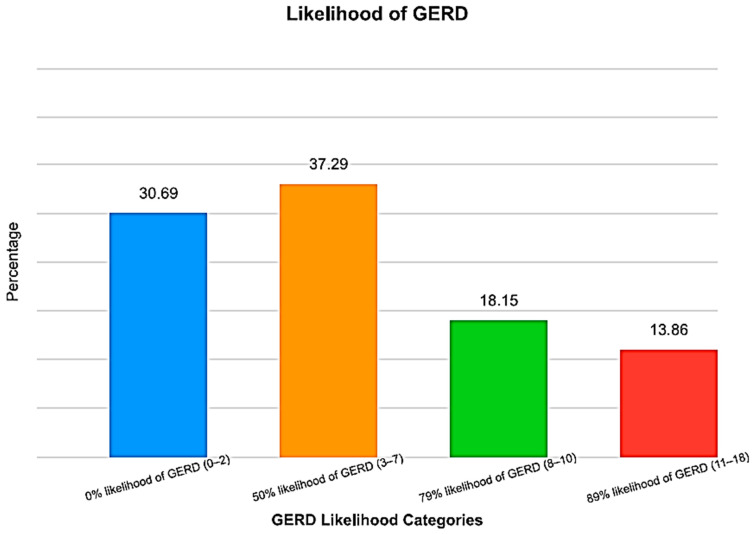
GERD likelihood distribution among participants (n = 303) in the GERMS project conducted in Saudi Arabia. Most participants had a 50% likelihood of GERD.

A total of 330 adults were recruited, of which 303 met the inclusion criteria. Thus, the response rate was 91.8%. Participants (n = 27) were excluded for reasons such as incomplete responses/unreachable by phone (n = 13), pregnancy (n = 3), liver cirrhosis (n = 3), renal impairment (n = 4), or being on dialysis (n = 4). The participants (n = 97; 32%) scored ≥ 8 in the GerdQ tool, with a mean score of 5.6 ± 4.3. Many participants (69.4%) had a ≥50% likelihood of GERD, with more than a third having a ≥79% likelihood (Figure 1). As expected, the mean GerdQ score increased with an increased likelihood, with the highest score obtained among the 89% likelihood group (Table 1).

Furthermore, the results showed that, on average, the participants with an 89% likelihood of GERD were middle-aged, with most aged between 40 and 59 years (Table 1). Furthermore, a significant proportion of adults with a 50% likelihood of gastroesophageal reflux disease (GERD) were aged 50–59 years, while the group with an 89% likelihood of GERD predominantly comprised individuals between 40 and 49 years old (*p* = 0.038; 95% CI = 0.24–12.44). Many women had an 89% likelihood of GERD compared to their counterparts (Table 1).

**Figure 2:**
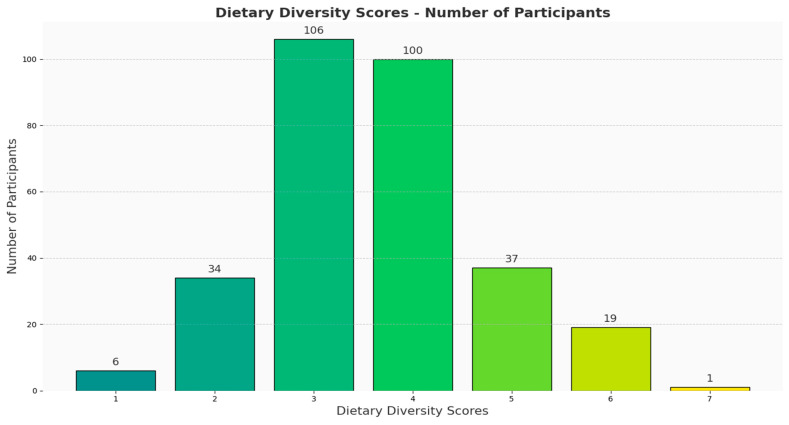
The distribution of participants’ dietary diversity scores (n = 303) in the GERMS project conducted in Saudi Arabia. The x-axis represents the scores, whereas the y-axis shows the number of participants. Most of the participants scored 3–4.

Moreover, many participants (n = 106, 35%) scored three on the Dietary Diversity Score Questionnaire, with very few (n = 57; 18.8%) scoring ≥ 5 (Figure 2). The total DDS did not differ significantly among the participants, irrespective of their GERD likelihood. In addition, very few participants were categorized as having a high level of dietary diversity (Table 2). Cereals ranked at the top of the list of plant-based foods consumed, whereas vitamin A-rich fruits and vegetables were at the bottom, irrespective of the patient’s likelihood of GERD. In addition, there were no significant differences in their food group intake, except for the legumes, nuts, and seeds group, whereby those with a lower likelihood of GERD had higher intakes from the legumes, nuts, and seeds group. Regarding animal-based foods, flesh meat and fish ranked highest among the consumed food groups, while organ meat was the least consumed. Also, the intake of milk and milk products differed significantly among the participants, with the lowest intake being reported by the 89% likelihood of GERD group (Table 2).

Many of the participants with a zero-percentage likelihood of GERD (*p* = 0.016; 95% CI = 0.03–0.43) and a 50% likelihood (*p* = 0.038; 95% CI = 0.01–0.39) had higher intakes of legumes, nuts, and seeds compared to their counterparts with a 79% likelihood of GERD. No statistically significant differences were observed between participants with 89% GERD likelihood and those in either the zero or 50% likelihood groups (*p* > 0.05). However, dairy consumption patterns demonstrated significant variation across GERD likelihood categories. Specifically, subjects with 50% GERD likelihood exhibited higher dairy product consumption than those with 89% likelihood (*p* = 0.041, 95% CI = 0.01–0.46). Similarly, participants in the 79% GERD likelihood category demonstrated significantly higher dairy product intake relative to the 89% likelihood group (*p* = 0.018, 95% CI = 0.04–0.56).

Using Pearson’s correlation, the results showed that GERD likelihood was negatively associated with age (r = −0.153; *p* = 0.008). As the intake of legumes, nuts, and seeds (r = 0.163; *p* = 0.005) increased, the likelihood of GERD decreased. In addition, as the intake of organ meat increased, so did the likelihood of GERD (r = 0.151, *p* = 0.009). In addition, DDSs increased significantly with the intake of other fruits and vegetables (r = 0.437; *p* = 0.0001). The intake of cereals and tubers (r = 0.114; *p* = 0.047) and legumes, nuts, and seeds (r = 0.231; *p* = 0.0001) increased significantly with the participants’ education level. When the participants were divided based on their GERD likelihood, the education level of the zero percentage likelihood participants was positively associated with the intake of legumes, nuts, and seeds (r = 0.382; *p* = 0.0001) and with the DDSs (0.329; *p* = 0.001). The intake of the latter foods correlated negatively with sex (r = −0.208; *p* = 0.045). Among the participants with a 50% likelihood of GERD, DDS was positively correlated with the consumption of legumes, nuts, and seeds (r = 0.232; *p* = 0.014) and with education level (r = 0.293; *p* = 0.002). In addition, episodes of regurgitation during the preceding week decreased significantly with a high intake of dark-green vegetables (r = −0.245; *p* = 0.009).

Moreover, among the participants with a 79% likelihood of GERD, higher education levels were associated with a low dark-green leafy vegetable intake (r = −0.313; *p* = 0.02). Their DDSs increased significantly with the increased consumption of dark-green leafy vegetables (r = 0.545; *p* = 0.0001) and legumes, nuts, and seeds (r = 0.667; *p* = 0.0001). On the other hand, among the participants with an 89% likelihood of GERD, legume, nut, and seed consumption was found to be inversely correlated with sex (r = −0.341; *p* = 0.027). The heartburn episodes of the same group directly correlated with education level (r = 0.344; *p* = 0.026).

The multiple regression analysis, with GERD likelihood as the dependent variable, demonstrated that legume, nut, and seed consumption was the strongest predictor, followed by female sex, age, and organ meat consumption. Analysis revealed that dairy product consumption demonstrated a modest but statistically significant protective effect against GERD (Table 3).

## 4. Discussion

Gastroesophageal reflux disease (GERD) is a common public health concern and a chronic gastrointestinal disorder that significantly impairs quality of life [28]. Our study showed that the consumption of legumes, nuts, and seeds correlated inversely with the likelihood of GERD. Thus, our hypothesis is accepted. Similarly, a systematic review revealed that the consumption of a Mediterranean diet rich in vegetables and fiber played a protective role against GERD [11,29]. Among adolescents, the consumption of the ‘dietary approaches to stop hypertension’ (DASH) diet, which is rich in fruits, vegetables, and legumes, was inversely associated with GERD and symptoms such as reflux and nausea [30].

Evidence shows that GERD is caused by oxidative stress [31], and legume consumers could be protected by the anti-inflammatory role that this food group plays [29]. Moreover, legumes are rich in fiber and can clean out gastric nitrites and reduce the nitric oxide concentration in the gastroesophageal junction, thus preventing gastric reflux [32]. However, it is important to note that although dietary fiber can decrease the number of gastroesophageal refluxes, it can sometimes increase their duration without significantly affecting gastric emptying and gastric acid secretion [33]. The evidence suggests that legumes, a significant source of plant-based protein, exhibit protective effects against gastroesophageal reflux. Clinical studies have demonstrated that legume consumption is inversely associated with acid reflux symptoms, specifically during the first hour post-consumption [34]. Moreover, research has established a positive correlation between legume intake and lower esophageal sphincter tone [35,36]. One possible explanation is that these foods are non-acidic and high in fiber, which may enhance their protective effect against GERD.

Moreover, our study showed that milk, milk products, and organ meat correlated differently with GERD likelihood. The former was negatively associated, whereas the latter was positively correlated with GERD. Previous studies reported that a higher fat meal content could be related to an increased acid exposure time in GERD patients compared to a low-fat meal [3]. A previous study in Saudi Arabia contradicted our findings. In that study, there was no relationship between GERD and fatty foods [17]. However, our study agreed with another study conducted in Germany, which reported that meat consumption positively correlated with increased reflux symptoms [32]. Organ meat is rich in fat and cholesterol, which are directly associated with an increased GERD risk.

Concerning demographic characteristics, our findings indicate that many women had an 89% likelihood of GERD (54.8%) compared to men (45.2%). Gender-specific variations observed in our study warrant cautious interpretation due to the relatively lower proportion of male participants in the study cohort. Our findings disagreed with a Korean study that found that non-erosive reflux disease was more prevalent among men than women [37]. It also disagreed with a previous study conducted in Saudi Arabia [19]. Contrary to our study, a previous American study showed no significant differences between men and women [38,39]. Other researchers agreed with our findings, stating that women were more susceptible to heartburn and acid regurgitation symptoms than their male counterparts [40,41,42]. In a quantitative esophageal symptom analysis, symptom frequency and severity were significantly higher among women than their male counterparts [43]. Possible explanations for women having a higher GERD likelihood could be related to their level of psychological stress and hormonal influences. For instance, estrogen and progesterone affect lower esophageal sphincter pressure and esophageal motility, increasing the reflux episodes among women. However, the current study did not investigate these factors.

Furthermore, as age increases, anatomical and physiological changes cause the degradation of the anti-reflux mechanisms at the gastroesophageal junction, increasing the risk of GERD [44]. However, that was not the case in our study, which showed an inverse association between age and the likelihood of GERD. Our findings agreed with those of Minatsuki et al., who found that a younger age was associated with non-erosive reflux disease [45]. Additionally, agreeing with our study, Pilotto et al. found that as people aged, they had a significantly decreased prevalence of heartburn and regurgitation. However, that study did not assess the severity and frequency of these symptoms [46].

The relationship between age and GERD presents conflicting evidence in the literature, with some studies reporting a positive correlation [22,47] and others indicating no significant association [48]. Our observation of an inverse relationship between age and GERD likelihood corresponds with the existing hypotheses that associate ageing with a decreased body mass, a diminished appetite, and increased comorbidities [46]. The observed age-related pattern in our study population may be attributed to differential stress exposure between the age groups, with younger individuals experiencing higher occupational and lifestyle-related stressors. In contrast, the older adults in our study population benefit from established social support systems and retirement benefits, potentially reducing stress-mediated GERD symptoms.

Interestingly, our findings revealed a significant direct relationship between education level and cereal, tuber, legume, nut, and seed intake and an inverse association with dark-green leafy vegetables. These findings are consistent with previous research, which showed that higher education levels were often linked to the high consumption of plant-based nutrient-dense foods [49,50,51]. In comparison, lower education levels were associated with poor-dietary patterns [52].

The current study is considered the first to be conducted in Al Madinah Al Munawarah, giving insight into GERD patients’ dietary quality and patterns. It highlighted crucial factors that require further investigation. In addition, the bias associated with data collection was kept to a minimum by providing extensive training for the nurses who conducted the face-to-face interviews. This helped reduce any bias related to missing data or skipping questions due to misunderstanding or unclarity. The tools used for data collection, namely IDDS and GerdQ, have been previously validated, increasing the credibility of the data obtained. The current paper reports part of the results from the GERMS project, and further findings will be published soon.

However, this study has some limitations that should be considered. Firstly, the sample was predominantly female, which could skew the results and potentially affect the inferences drawn from the findings. In addition, the sampling method was not randomized, making it difficult to generalize the findings to the population of Al Madinah Al Munawarah. One of these limitations is related to using a single 24 h recall record for the IDDS, which might have introduced bias due to the day-to-day variations in intakes. However, the reliability of a one-day IDDS was high, and the findings could be valid and reflective of the situation. The disadvantage of using 24 h recall records, in general, is that this method depends on memory. As mentioned earlier in the Methods Section, the manner in which the method was executed placed a minimal burden on the participants’ memory, thereby enhancing the likelihood of accurate food recall.

Furthermore, several vegetarian diets are associated with reduced oxidative stress and increased health benefits. In the current study, we used the IDDS tool to assess the quality and diversity of diets. However, the IDDS lists foods that cannot be modified. In addition, the cross-sectional study design has limitations, limiting the causal inferences, and the protective effect of the intake of PBFs could not be deducted entirely by a cross-sectional study design. Longitudinal follow-up studies would be optimal to assess the actual GERD risk development among participants over time.

The current study included only a few demographic characteristics, such as age and education level. It would have been ideal to include other factors, e.g., obesity, a previous diagnosis of GERD, abdominal surgery, and other relevant factors. However, the current study focused mainly on plant-based and animal-based diets and their relationship with the likelihood of GERD, which is why only a few demographic characteristics were included.

## 5. Conclusions

Our findings suggest an inverse relationship between the intake of plant-based food, namely legumes, nuts, and seeds, and milk and milk products with the likelihood of GERD. This finding indicates that these foods could protect against the disease. In addition, being middle-aged, being a woman, and consuming animal-based food, namely organ meat, were directly associated with an increased likelihood of GERD. In addition, a high education level is correlated with consuming dark-green vegetables. This study has several recommendations; a large study with a randomized sampling method and a representative population is encouraged in the future. A longitudinal study design is recommended to follow up on the development of GERD based on the consumption of PBFs and demographic characteristics. Targeted GERD intervention protocols should prioritize female populations, individuals with limited educational attainment, and subjects demonstrating suboptimal dietary patterns—specifically, those characterized by low consumption of legumes, nuts, seeds, milk, and milk products, along with high consumption of organ meats.

## Figures and Tables

**Table 1:** Demographic characteristics of participants, as categorized by GERD likelihood (n = 303).

Parameters	0% Likelihood n = 93 (30.7%)	50% Likelihood n = 113 (37.3%)	79% Likelihood n = 55 (18.2%)	89% Likelihood n = 42 (13.8%)	Total n = 303 (100%)	*p*-Value
**Age (years)**						0.026 ^§^
Mean (SD)	52.1 (1.7)	52.6 (11.8)	49.0 (14.6)	46.2 (12.8)	51 (12.8)	0.008
18–28	4 (4.3)	6 (5.3)	7 (12.7)	5 (12.0)	21 (7.0)	
29–39	8 (8.6)	7 (6.2)	4 (7.3)	6 (14.3)	25 (8.3)	
40–49	27 (29)	23 (20.4)	12 (21.8)	14 (33.3)	77 (25.4)	
50–59	25 (27)	47 (41.6)	21 (38.0)	12 (28.6)	105 (34.7)	
60–69	20 (21.5)	23 (20.4)	5 (9.0)	3 (7.0)	51 (16.8)	
≥70	9 (9.7)	7 (6.2)	6 (11.0)	2 (4.8)	24 (8.0)	
**Sex (numbers and percentages)**						0.012
Men	26 (28.0)	27 (24.0)	23 (41.8)	19 (45.2)	95 (31.4)	
Women	67 (72.0)	86 (76.0)	32 (58.2)	23 (54.8)	208 (68.6)	
**Education level (numbers and percentages)**						0.973
Low education ^a^	82 (88.0)	99 (87.6)	45 (81.8)	35 (83.3)	261 (86.0)	
High education ^b^	11 (12.0)	14 (12.4)	10 (18.2)	7 (16.7)	42 (14.0)	
**GerdQ score (mean and SEM)**						0.0001
Total GerdQ score	0.91 (0.1)	4.96 (0.1)	8.89 (0.1)	13.3 (0.3)	5.59 (0.2)	

^a^ Low education levels include no formal schooling, religious schools, elementary schooling, or secondary schooling; ^b^ high education levels include university degrees and higher; *p*-value obtained from the Chi-square test; and ^§^ *p*-value obtained from Tukey’s HSD post hoc ANOVA. SEM stands for standard error of the mean.

**Table 2:** The intake of food groups, as categorized by GERD likelihood (n = 303).

	0% Likelihood n = 93 (30.7%)	50% Likelihood n = 113 (37.3%)	79% Likelihood n = 55 (18.2%)	89% Likelihood n = 42 (13.8%)	Total n = 303 (100%)	*p*-Value
**Dietary Diversity Scores (numbers and percentages)**						0.654 ^§^
Mean (SD)	3.63 (1.2)	3.71 (1.1)	3.49 (0.9)	3.55 (1.1)	3.62 (1.1)	
**Dietary Diversity Category**						0.626
Low (≤3)	45 (48.4)	54 (47.8)	30 (54.5)	17 (40.5)	146 (48.2)	
Medium (4–5)	40 (43.0)	51 (45.0)	23 (41.8)	23 (54.8)	137 (45.2)	
High (≥6)	8 (8.6)	8 (7.0)	2 (3.6)	2 (4.8)	20 (6.6)	
**Plant-Based Food Groups (numbers and percentages)**						
Cereals and tubers	91 (97.8)	110 (97.3)	52 (94.5)	42 (100)	295 (97.4)	0.939
Vitamin A-rich fruit and vegetables	1 (1.1)	1 (0.9)	0	0	2 (0.7)	0.361
Dark-green leafy vegetables	22 (23.7)	26 (23.0)	16 (29.0)	10 (23.8)	74 (24.4)	0.715
Other fruits and vegetables	54 (58.1)	77 (68.0)	34 (61.8)	26 (62.0)	191 (63.0)	0.724
Legumes, nuts, and seeds	35 (37.6)	39 (34.5)	8 (14.5)	9 (21.4)	91 (30.0)	0.005
**Animal-Based Foods Groups (numbers and percentages)**						
Organ meat	0	2 (1.8)	2 (3.6)	3 (7.1)	7 (2.3)	0.068
Flesh meat/fish	65 (70)	71 (62.8)	32 (58.2)	34 (81.0)	202 (66.7)	0.078
Eggs	18 (19.4)	26 (23.0)	12 (21.8)	10 (23.8)	66 (21.8)	0.915
Milk and milk products	54 (58.1)	67 (59.3)	36 (65.5)	15 (35.7)	172 (56.7)	0.022

*p*-value obtained from the Chi-square test; ^§^ *p*-value obtained from post hoc ANOVA; and the values are for the participants who stated that they consumed the foods from these food groups. The dietary intake analysis showed that participants with a 89% GERD likelihood had significantly lower legumes, nuts, seeds, and dairy products consumption than the other groups.

**Table 3:** Multiple Regression Model: Dietary and Demographic Determinants of GERD Likelihood.

Variables	B	Standard Error	Beta	t	Significance Level
Age	−0.012	0.004	−0.155	−2.705	0.007
Sex	−0.360	0.126	−0.165	−2.849	0.005
Education level	−0.011	0.041	−0.017	−0.273	0.785
Cereals and Tubers	−0.019	0.353	−0.003	−0.053	0.958
Vitamin A-rich fruits and vegetables	−0.498	0.696	−0.040	−0.716	0.474
Dark-green leafy vegetables	0.039	0.139	0.017	0.281	0.779
Other fruits and vegetables	0.013	0.124	0.006	0.106	0.916
Legumes, nuts and seeds	−0.375	0.131	−0.170	−2.863	0.005
Organ meat	0.866	0.375	0.129	2.310	0.022
Milk and milk products	−0.231	0.117	−0.113	−1.978	0.049

Adjusted R^2^ = 33.249; Residue = 274.436; F = 3.526; and *p* < 0.001.

## Data Availability

Due to privacy and ethical issues, the data presented in this study are available upon request from the corresponding author.

## Supplementary Materials

The following supporting information can be downloaded at: https://www.mdpi.com/article/10.3390/ijerph21121696/s1, Table S1: GerdQ Questionnaire for the determination of GERD likelihood; Table S2: The Dietary Diversity Score Scale for the determination of the quality of diet.

## Abbreviations

CI	Confidence interval
IDDS	Individual Dietary Diversity Score
GERD	Gastroesophageal reflux disease
GERMS	Gastroesophageal Reflux Madinah Study
PBF	Plant-based food
