# Supplementary material for: Associations of Plant-Based Foods, Animal Products, and Selected Sociodemographic Factors with Gastroesophageal Reflux Disease Risk
Source: Int J Environ Res Public Health. 2024 Dec 19;21(12):1696. doi: 10.3390/ijerph21121696 (PMC11728439; doi:10.3390/ijerph21121696)
Supplement: Supplementary file 1 [file ijerph-21-01696-s001.zip › ijerph-3282644-supplementary.pdf]

Supplementary Materials

Table S1. GerdQ Questionnaire for the determination of GERD likelihood.

Question	Frequency Score (Points) for Symptom			
	**0 day**	**1 day**	**2–3 days**	**4–7 days**
How often did you have a burning feeling behind your breastbone (heartburn)?	0	1	2	3
How often did you have stomach contents (liquid or food) moving upwards to your throat or mouth (regurgitation)?	0	1	2	3
How often did you have pain in the center of the upper stomach?	3	2	1	0
How often did you have nausea?	3	2	1	0
How often did you have difficulty getting a good night's sleep because of your heartburn and/or regurgitation?	0	1	2	3
How often did you take additional medication for your heartburn and/or regurgitation, other than what the physician told you to take (such as Tums, Rolaids, Maalox)?	0	1	2	3

Table S2. The Dietary Diversity Score Scale for the determination of the quality of diet.

DIETARY DIVERSITY QUESTIONARE [1]

Please describe the foods (meals and snacks) that you ate yesterday during the day and night, whether at home or outside the home. Start with the first food eaten in the morning.
Write down all food and drinks mentioned by the respondent. When the respondent has finished, probe for meals and snacks not mentioned.

Breakfast	Snack	Lunch	Snack	Dinner	Snack

[Household level: consider foods eaten by any member of the household, and exclude foods purchased and eaten outside of the home]

When the respondent recall is complete, fill in the food groups based on the information recorded above. For any food groups not mentioned, ask the respondent if a food item from this group was consumed.

Question Number	Food Group	Examples	YES = 1<br>No = 0
1	CEREALS		
2	VITAMIN A RICH VEGETABLES AND TUBERS		
3	WHITE TUBERS AND ROOTS		
4	DARK GREEN LEAFY VEGETABLES		
5	OTHER VEGETABLES		
6	VITAMIN A RICH FRUITS		
7	OTHER FRUITS		
8	ORGAN MEAT (IRON-RICH)		
9	FLESH MEATS		
10	EGGS		
11	FISH		
12	LEGUMES, NUTS AND SEEDS		
13	MILK AND MILK PRODUCTS		
14	OILS AND FATS		
15	RED PALM PRODUCTS		
16	SWEETS		
17	SPICES, CONDIMENTS, BEVETAGES		
			**YES = 1<br>No = 0**
Individual level only	Did you eat anything (meal or snack) OUTSIDE of the home yesterday?		
Household level only	Did you or anyone in your household eat anything (meal or snack) OUTSIDE of the home yesterday?		
